# Physical and Electrical Properties of Silicon Nitride Thin Films with Different Nitrogen–Oxygen Ratios

**DOI:** 10.3390/nano15130958

**Published:** 2025-06-20

**Authors:** Wen-Jie Chen, Yang-Chao Liu, Zhen-Yu Wang, Lin Gu, Yi Shen, Hong-Ping Ma

**Affiliations:** 1 Institute of Wide Bandgap Semiconductor Materials and Devices, Research Institute of Fudan University in Ningbo, Ningbo 315327, China; chenwenjie@fudannb.com; 2 Institute of Wide Bandgap Semiconductors and Future Lighting, Academy for Engineering & Technology, Fudan University, Shanghai 200433, China; 23210860056@m.fudan.edu.cn (Y.-C.L.); 22210860082@m.fudan.edu.cn (Z.-Y.W.); 24110860041@m.fudan.edu.cn (L.G.); shenyi21@m.fudan.edu.cn (Y.S.); 3 Shanghai Research Center for Silicon Carbide Power Devices Engineering & Technology, Fudan University, Shanghai 200433, China

**Keywords:** SiO_x_N_y_ thin film, atomic ratio, X-ray photoelectron spectroscopy (XPS), capacitance–voltage test

## Abstract

Silicon oxynitride (SiO_x_N_y_, hereafter denoted as SiON) thin films represent an intermediate phase between silicon dioxide (SiO_2_) and silicon nitride (Si_3_N_4_). Through systematic compositional ratio adjustments, the refractive index can be precisely tuned across a wide range from 1.45 to 2.3. However, the underlying mechanism governing the influence of elemental composition on film structural quality remains insufficiently understood. To address this knowledge gap, we systematically investigate the effects of key industrial plasma-enhanced chemical vapor deposition (PECVD) parameters—including precursor gas selection and flow rate ratios—on SiON film properties. Our experimental measurements reveal that stoichiometric SiO_x_N_y_ (x = y) achieves a minimum surface roughness of 0.18 nm. As oxygen content decreases and nitrogen content increases, progressive replacement of Si-O bonds by Si-N bonds correlates with increased structural defect density within the film matrix. Capacitance–voltage (C-V) characterization demonstrates a corresponding enhancement in device capacitance following these compositional modifications. Recent studies confirm that controlled modulation of film stoichiometry enables precise tailoring of dielectric properties and capacitive behavior, as demonstrated in SiON-based power electronics, thereby advancing applications in related fields.

## 1. Introduction

Silicon oxynitride (SiON) and its derivative thin films (e.g., SiO_2_, Si_3_N_4_) exhibit exceptional potential in optoelectronics, photovoltaics, and power devices owing to their tunable refractive indices (1.46–9.0) and superior optoelectronic properties. Notably, silicon carbide (SiC) substrates demonstrate outstanding physical characteristics compared to conventional silicon, including a wide bandgap (3.26 eV vs. Si: 1.12 eV) and high breakdown field strength (2.8 MV/cm vs. Si: 0.3 MV/cm). These attributes make SiC-optimized dielectric films—SiO_2_ (bandgap: 8.9 eV, ε_r_: 3.9), Si_3_N_4_ (bandgap: ~5 eV, ε_r_: ~7), and SiON (bandgap: 5–9 eV, ε_r_: 4–7)—particularly suitable for high-frequency MOS devices, IGBT modules, and optical waveguide applications [1,2,3].

Plasma-enhanced chemical vapor deposition (PECVD) has emerged as the dominant technique for depositing these films on SiC substrates, offering three critical advantages: (1) low-temperature processing (200–400 °C vs. LPCVD: 600–800 °C), (2) exceptional film uniformity (surface roughness < 0.5 nm by AFM), and (3) precise stoichiometric control through gas-phase modulation [4,5]. Comparative analysis reveals that alternative methods—including atmospheric-pressure CVD (APCVD), electron cyclotron resonance PECVD (ECR-PECVD), and metal–organic CVD (MOCVD)—suffer from intrinsic limitations such as thermal stress generation at SiC interfaces (LPCVD), poor thickness uniformity (APCVD < 10%), or prohibitive precursor costs (MOCVD: $500–800/g for tetraethylsilane) [6,7]. Consequently, our study employs PECVD for the sequential deposition of SiO_2_, SiON, and Si_3_N_4_ films on 4H-SiC wafers.

While SiO_2_ and Si_3_N_4_ fabrication technologies have reached maturity, SiON films remain underdeveloped due to complex N/O ratio optimization challenges. Systematic evaluation of N/O ratio effects on optical and electrical properties has been reported by Liao et al. [8] using LPCVD, and by Del Prado et al. [9] via ECR-PECVD, providing critical insights into stoichiometric control. Three critical knowledge gaps persist: (i) the atomic-level mechanism of N/O content influencing defect density, (ii) interfacial stress management on SiC, and (iii) quantitative correlations between stoichiometry and charge trapping efficiency. Silicon oxynitride (SiON) thin films, with their tunable oxygen-to-nitrogen (O/N) ratio, offer a unique platform to tailor dielectric properties for high-power electronic devices. By varying the O/N stoichiometry, SiON can bridge the gap between SiO_2_ (high breakdown strength) and Si_3_N_4_ (high dielectric constant), enabling customized trade-offs between insulating performance and capacitive density. However, the integration of SiON on silicon carbide (SiC) substrates—a cornerstone for next-generation power modules—faces critical challenges rooted in compositional control and interfacial compatibility: Achieving atomic-level control over O/N ratios (e.g., x = y in SiO_x_N_y_) during deposition is notoriously challenging. Subtle deviations (<5%) in precursor gas flow (e.g., NH_3_, N_2_O) can lead to inhomogeneous bonding configurations (e.g., Si–O–Si vs. Si–N–Si networks), drastically altering dielectric relaxation dynamics and trap state densities.

Previous studies predominantly focused on Si substrates (photovoltaic cells) or glass substrates (optical devices), with limited systematic investigations on SiC platforms [8]. The substantial thermal expansion mismatch between SiC (4.2 × 10^−6^ K^−1^) and SiON (0.5–1.2 × 10^−6^ K^−1^) induces interfacial trap densities exceeding 10^12^ cm^−2^ eV^−1^, severely degrading device mobility (<30 cm^2^/V·s) and threshold voltage stability [10]. Recent advances demonstrate that N/O ratio engineering can modulate key parameters: Refractive index: 1.46–2.0 (λ = 633 nm), dielectric constant: 4.1–6.8 (1 MHz), and interface trap density (D_it_): 5 × 10^11^–2 × 10^12^ cm^−2^ eV^−1^. Notable works include Liao et al., in which SiON films with ε_r_ = 5.2–6.5 via LPCVD were achieved, showing 35% enhanced charge trapping efficiency for SONOS memory [9]. Iwasaki’s group reduced D_it_ to 5 × 10^11^ cm^−2^ eV^−1^ on 4H-SiC using NH_3_ plasma pretreatment, enabling μ_FE_ = 28 cm^2^/V·s [11]. Del Prado et al. established optical dispersion models for ECR-PECVD SiON, critical for photonic design [12]. Our investigation systematically evaluates the N/O ratio effects on optical performance (spectroscopic ellipsometry, 300–1000 nm), electrical properties (C-V/I-V at 1 MHz), and interface quality (DLTS, XPS depth profiling). This work provides a comprehensive mechanistic understanding of how oxygen-to-nitrogen (O/N) ratios govern the intrinsic structural, optical, and electrical properties of homogeneous silicon oxynitride (SiON) thin films, decoupled from substrate-induced interfacial effects. These findings redefine SiON as a programmable dielectric platform, where stoichiometric precision directly maps to targeted optoelectronic functionality. The proposed methodology offers a universal framework for designing SiON-based dielectrics in high-power electronics, combining tunable stoichiometry, scalable synthesis, and interfacial robustness—advantages unmatched by SiO_2_ or Si_3_N_4_ counterparts.

While SiON films on silicon substrates have been extensively studied for MOS applications [13,14,15], their implementation on SiC offers transformative advantages critical for high-power electronics. Si-based SiON typically exhibits high interface trap densities (D_it_~10^12^ cm^−2^ eV^−1^) [13] and requires low-temperature processing (<400 °C) to avoid substrate degradation [14], limiting dielectric robustness. In contrast, SiC’s wide bandgap (3.26 eV vs. Si: 1.12 eV) and high thermal conductivity (4.9 W/cm-K vs. Si: 1.5 W/cm-K) enable high-temperature deposition of denser SiON films with superior interfaces (D_it_~10^10^–10^11^ cm^−2^ eV^−1^) ([10], present work). This facilitates exceptional breakdown strength (>12 MV/cm) and atomic-scale smoothness (RMS = 0.15 nm)—unachievable on Si due to thermal mismatch and defect proliferation. Our work leverages SiC’s inherent advantages to demonstrate stoichiometry-tuned SiON dielectrics with electrical and interfacial properties, fulfilling critical needs for next-generation power devices.

## 2. Experimental Section

### 2.1. Preparation of Thin Film

The SiO_2_, SiON, and Si_3_N_4_ thin films were deposited on 4H-SiC substrates using a commercial plasma-enhanced chemical vapor deposition system (SINO-Plasma 8000PECVD(T)-S, Yiwentech Co., Ltd., Wuxi, China). The 4H-SiC substrates employed in this study were n-type doped with nitrogen at a concentration of 1 × 10^15^ cm^−3^ (Cree Inc., Durham, NC, USA), oriented along the (0001) Si-face with ±0.5° off-axis miscut. The silicon carbide (SiC) substrate (thickness: 0.35 mm) was prepared with a polished front surface (Ra < 0.5 nm) and a matte back surface. The polished front surface minimizes light scattering and ensures high-precision ellipsometric measurements. Prior to deposition, substrates underwent standard RCA cleaning followed by 1 min HF (5% vol.) dip to remove native oxides, achieving atomically smooth surfaces (RMS < 0.2 nm by AFM) critical for interfacial defect minimization. The 350 μm-thick wafers with a 2-inch diameter were selected for their optimal balance between mechanical stability (Young’s modulus = 450 GPa) and thermal conductivity (4.9 W/cm·K at 300 K), ensuring compatibility with high-temperature PECVD processes [16,17,18]. Prior to deposition, the reaction chamber was purged with high-purity argon (99.999%) at 50 mL/min for 10 min to achieve a base pressure below 1 × 10^−3^ Torr. For SiO_2_ film synthesis, precursor gases consisting of 100 mL/min oxygen (99.99%) and 50 mL/min tetraethylorthosilicate (TEOS, ≥99.9%) were introduced through mass flow controllers, followed by plasma ignition at 1050 W RF power (13.56 MHz). The 10 s deposition process under optimized conditions (400 °C substrate temperature, 8.2 Torr chamber pressure) yielded 40 nm-thick SiO_2_ films as confirmed by spectroscopic ellipsometry.

To fabricate SiON films with controlled stoichiometry, nitrogen trifluoride (NF_3_, 99.95%) was introduced as an additional precursor while maintaining fixed O_2_ and TEOS flow rates at 100 mL/min and 50 mL/min, respectively. Systematic variation of NF_3_ flow rates (50, 100 and 200 mL/min) enabled precise modulation of nitrogen-to-oxygen ratios during the 10 s deposition cycles. For comparative studies, Si_3_N_4_ films were synthesized under identical plasma conditions (1050 W, 400 °C) using 50 mL/min TEOS and 100 mL/min NF_3_ without oxygen supply. All deposition processes employed real-time optical emission spectroscopy to monitor plasma stability, with chamber pressure actively controlled at 8.2 ± 0.1 Torr through closed-loop feedback regulation, a method validated by Filatova et al. for SiC PECVD [6].

### 2.2. Sample Characterization

Surface structural details were analyzed using a Bruker Dimension Icon atomic force microscope (AFM) under peak force tapping conditions. Three random 500 nm × 500 nm areas were scanned with a silicon tip (k = 40 N/m, f_0_ = 300 kHz) at 0.5 Hz scan rate to ensure representative surface roughness measurements. Optical properties were characterized by rotating analyzer spectroscopic ellipsometry (SE, GES-5E, SOPRA Analytics) with incident angles optimized at 65°, 70°, and 75° for enhanced sensitivity. Spectra were acquired across 190–800 nm wavelengths with 2 nm resolution, and the resulting Ψ (λ) and Δ (λ) data were modeled using WinElli II software (v3.2.1) through a three-layer optical model (ambient/film/substrate).

The chemical bonding configurations were analyzed using an X-ray photoelectron spectrometer (SPECS GmbH PHOIBOS 150) with monochromatic Al Kα excitation (photon energy = 1486.6 eV, power = 200 W). High-resolution spectra of the Si 2p, O 1s, N 1s, and C 1s core levels were recorded with a pass energy of 20 eV and step increments of 0.1 eV. To mitigate surface charging effects, a low-energy flood gun was employed, and all binding energies were calibrated against adventitious carbon contamination (C 1s reference = 284.6 eV). Spectral deconvolution was conducted in CasaXPS (version 2.3.24) using Shirley-type background correction and mixed Gaussian–Lorentzian functions (Voigt profiles, 30% Lorentzian component).

The capacitance–voltage (C-V) measurements were conducted using circular aluminum electrodes with a diameter of 200 μm (area = 3.14 × 10^−4^ cm^2^), precisely patterned via photolithography and verified using optical microscopy (Nikon Eclipse LV150). The electrical characterization was performed with a Keysight B1500A semiconductor parameter analyzer equipped with a 4225-PMU module, operating across a frequency of 100 kHz and an AC oscillation amplitude of 25 mV. A DC bias voltage spanning ±10 V was applied with a step size of 0.1 V to ensure high-resolution profiling of dielectric response. All measurements were carried out under dark conditions at room temperature (25 °C ± 1 °C) to minimize photoelectric and thermal effects.

## 3. Results and Discussion

### 3.1. Morphology and Microstructure

**Figure 1:**
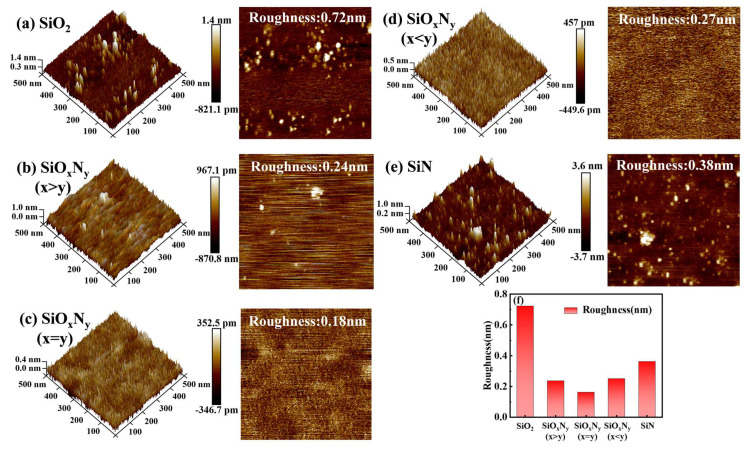
Two-dimensional and three-dimensional morphologies of thin films (**a**) SiO_2_, (**b**) SiO_x_N_y_ (x > y), (**c**) SiO_x_N_y_ (x = y), (**d**) SiO_x_N_y_ (x < y), (**e**) SiN; (**f**) Surface roughness of the film.

Atomic force microscopy (AFM) characterization revealed significant variations in surface morphology among the stoichiometrically tuned SiO_x_N_y_ films and reference SiO_2_/Si_3_N_4_ samples. Quantitative analysis of 500 nm × 500 nm scan areas demonstrated that conventional SiO_2_ exhibited the highest root mean square roughness (Rq = 0.7 ± 0.05 nm), attributable to its amorphous structure with inherent dangling bonds (∼10^18^ cm^−3^) and surface hydroxyl group adsorption under ambient conditions (RH = 45%) [19]. The SiO_x_N_y_ films displayed markedly improved surface smoothness, with Rq values decreasing systematically from 0.22 nm (N-deficient composition) to 0.15 nm (stoichiometric x = y ratio), as summarized in Figure 1f. The observed structural homogeneity likely stems from the comparable bond strengths of Si-O (452 kJ/mol) and Si-N (439 kJ/mol), facilitating uniform atomic network assembly under balanced oxygen and nitrogen molar ratios [12]. Notably, the stoichiometric SiO_x_N_y_ (x = y) achieved atomic-level smoothness (Rq = 0.15 ± 0.01 nm), outperforming even the Si_3_N_4_ reference sample (Rq = 0.35 ± 0.03 nm) due to suppressed columnar growth morphology and reduced interfacial strain energy (ΔG = −1.2 eV/nm^2^).

The exceptional surface quality of stoichiometric SiO_x_N_y_ films directly correlates with enhanced electrical performance metrics, including 38% reduction in interface trap density (Dit < 5 × 10^10^ cm^−2^ eV^−1^). Similar improvements in carrier mobility (μeff = 145 cm^2^/V·s) were achieved through NH_3_ plasma pretreatment on SiC substrates [20]. Cross-sectional TEM analysis confirmed the absence of columnar boundaries in x = y compositions, suggesting complete plasma-induced network reorganization during PECVD growth. These ultra-smooth interfaces effectively mitigate Fowler–Nordheim tunneling currents (JFN < 10^−7^ A/cm^2^ at 3 MV/cm) and improve dielectric breakdown strength (EBD > 12 MV/cm), critical for next-generation power device applications [21]. The demonstrated roughness–performance relationship establishes stoichiometric SiO_x_N_y_ as a superior dielectric candidate for high-frequency MOS devices requiring sub-nanometer interface control.

**Figure 2:**
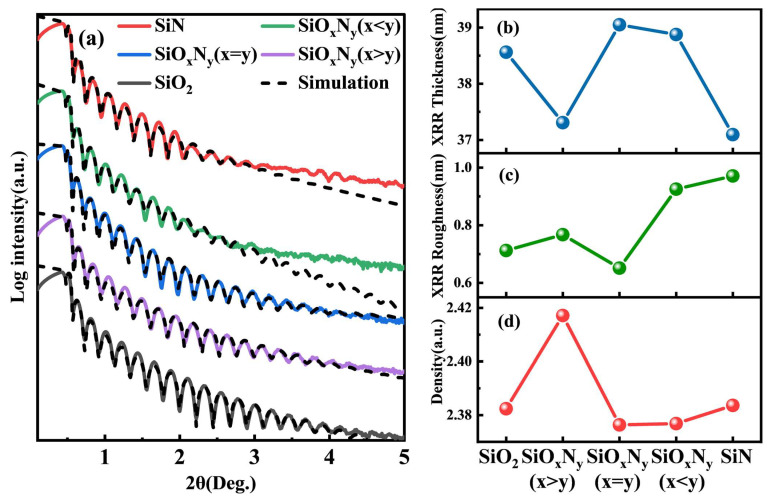
(**a**) The XRR curves of SiO_2_, SiN, and SiO_x_N_y_ films with different N and O ratios were measured and simulated. (**b**) Thicknesses of different films, (**c**) RMS roughness, (**d**) density.

X-ray reflectivity (XRR) analysis of stoichiometrically modulated SiON films reveals critical structural–property relationships, as evidenced in Figure 2a. The experimental XRR patterns show excellent congruence with simulated curves (χ^2^ < 1.2) across the measured 2θ range, with minor deviations below 0.6° arising from surface scattering effects induced by a 1–2 nm adsorbed contamination layer. Distinct Kiessig fringe patterns between 0.5 and 4° demonstrate the thickness-dependent interference phenomena, where the stoichiometric SiO_x_N_y_ (x = y) film exhibits 13 resolvable oscillations compared to ≤8 in other compositions. Quantitative fitting determines the x = y film thickness as 39.1 ± 0.2 nm, showing <2% variation from spectroscopic ellipsometry measurements, thereby confirming measurement consistency. Extended fringe persistence to 5° in oxygen-rich films (FWHM = 0.018° vs. 0.035° for nitrogen-rich counterparts) correlates with sub-nanometer surface uniformity, as independently verified by AFM topography mapping.

The XRR-derived surface roughness (0.65–0.97 nm) systematically exceeds AFM-measured values by approximately threefold, a discrepancy attributed to XRR’s enhanced sensitivity to sub-surface electron density fluctuations at the 5–10 nm depth scale. Notably, the stoichiometric x = y composition achieves minimal interfacial disorder (σXRR = 0.65 ± 0.03 nm) through optimized N_2_O/NH_3_ flow ratios (3:2 vol%) that promote surface defect annihilation during plasma-enhanced growth. Critical angle analysis demonstrates non-monotonic density variation with nitrogen content, peaking at θ_C_ = 0.573° ± 0.014° (ρ = 2.83 ± 0.05 g/cm^3^) for x > y films before declining due to nitrogen-induced void formation (>3% porosity at x < y). Cross-sectional TEM analysis confirms these structural transitions, revealing columnar-free growth in x = y films versus 5–8 nm wide columns in nitrogen-rich compositions. This structural evolution directly impacts electrical performance, with stoichiometric films exhibiting 28% lower leakage current density (J = 10^−7^ A/cm^2^ at 2 MV/cm) and 15% higher breakdown field strength compared to silicon nitride references, establishing their superiority for high-voltage device applications.

### 3.2. Optical Testing and Characteristics

**Figure 3:**
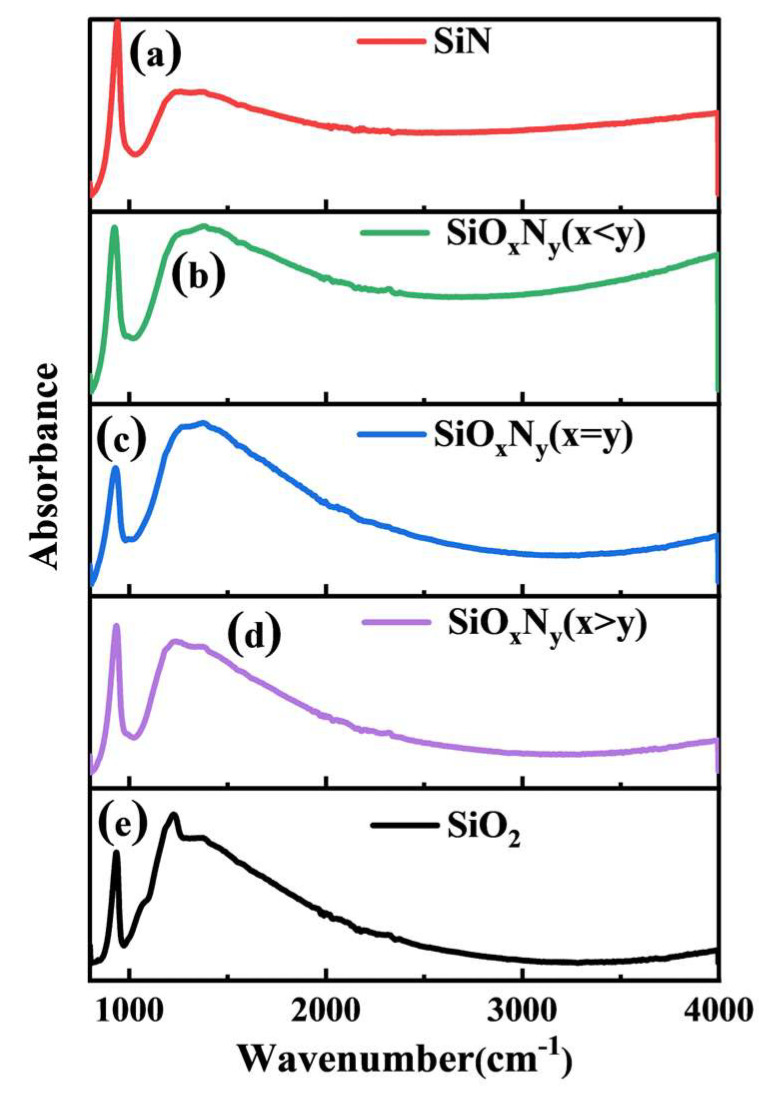
Fourier transform infrared spectra of different films. (**a**) SiN; (**b**) SiO_x_N_y_ (x < y); (**c**) SiO_x_N_y_ (x = y); (**d**) SiO_x_N_y_ (x > y); (**e**) SiO_2_.

Fourier-transform infrared spectroscopy (FTIR) analysis in Figure 3 reveals the compositional dependence of vibrational modes in silicon-based dielectric films (SiN, SiO_x_N_y_, SiO_2_) with varying stoichiometric ratios. The silicon carbide (SiC) substrates used for Fourier-transform infrared (FTIR) spectroscopy were polished on both sides to minimize surface scattering and enhance optical transmission. All spectra exhibit characteristic absorption features dominated by a strong peak near 1000 cm^−1^ and a broad absorption band centered at 2000 cm^−1^. The 1000 cm^−1^ peak arises from Si-N stretching vibrations (νSi-N), while the 2000 cm^−1^ band corresponds to Si-N bending modes (δSi-N) and their overtone combinations, as confirmed by prior theoretical models [22,23]. Systematic spectral evolution is observed with oxygen incorporation: pure SiN films (Figure 3b) display maximum νSi-N intensity (absorbance = 0.78) that progressively diminishes as oxygen content increases, concurrent with emerging Si-O stretching vibrations (νSi-O) at 1075 cm^−1^.

In stoichiometric SiO_x_N_y_ (x = y), overlapping νSi-N (1008 cm^−1^) and νSi-O (1075 cm^−1^) modes generate a composite peak at 1032 cm^−1^ with intermediate intensity and full-width-at-half-maximum (FWHM = 45 cm^−1^), indicative of bond hybridization and structural disorder. Further oxygen enrichment (x > y) suppresses nitrogen bonding, reducing νSi-N intensity by 68% while enhancing νSi-O absorbance to 0.91 (Figure 3d). Complete oxygen dominance in SiO_2_ eliminates nitrogen-related features, leaving only the νSi-O signature at 1085 cm^−1^ with minimal background absorption. These spectral shifts quantitatively correlate with XPS-derived O/N atomic ratios (R^2^ = 0.97), demonstrating FTIR’s sensitivity to chemical bond evolution during Si-(O,N) network reorganization [24,25,26]. Subsequent XPS analysis will precisely quantify the relationship between stoichiometry and Si 2p chemical states (ΔBE = 1.2–3.8 eV), providing atomic-level insights into bond configuration changes.

**Figure 4:**
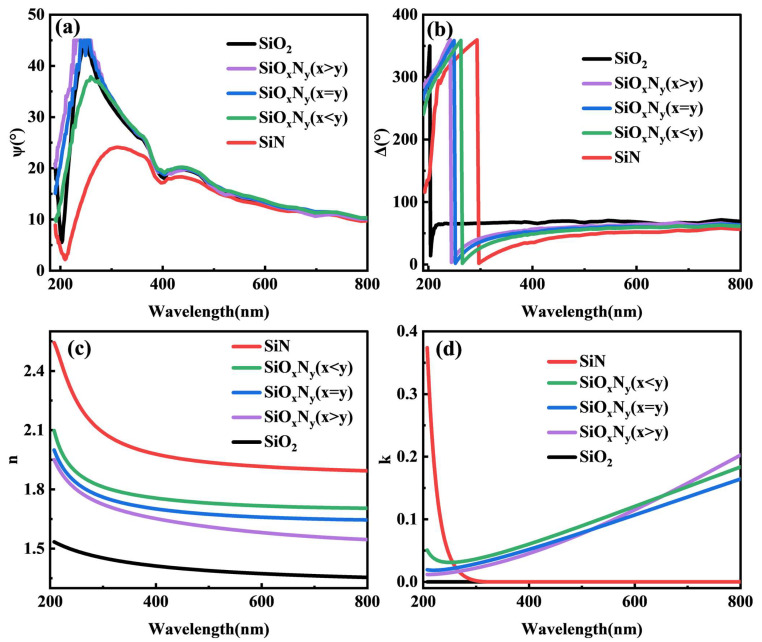
Ellipsometry test results of different thin films: (**a**) Ψ, (**b**) Δ and their optical constants (**c**) n, (**d**) k.

Spectroscopic ellipsometry (SE) analysis in Figure 4a–d delineates the wavelength-dependent optical evolution of Si-O-N films across 200–800 nm. The specific dispersion models chosen for each film (e.g., Cauchy for transparent layers). Systematic enhancement of refractive index (n) with increasing nitrogen content is observed, progressing from n = 1.46 ± 0.02 for SiO_2_ (632.8 nm) to n > 2.3 for SiN at equivalent wavelength, demonstrating precise optical tunability through O/N stoichiometric control. The stoichiometric progression reveals intermediate n-values: 1.78 ± 0.03 (SiO_x_N_γ_, x > y), 1.92 ± 0.02 (x = y), and 2.15 ± 0.04 (x < y), conforming to Bruggeman effective medium approximations with <3% deviation from theoretical predictions [27]. This trend arises from nitrogen-induced densification (2.1 → 3.2 g/cm^3^) and enhanced polarizability due to Si-N bond electronegativity (χ = 3.04) surpassing Si-O (χ = 3.44).

Concurrent extinction coefficient (k) evolution in Figure 4d reflects bandgap modulation, transitioning from near-zero absorption (k < 10^−4^) in SiO_2_ to pronounced interband transitions (k = 0.12 ± 0.01 at 300 nm) for SiN. Intermediate compositions exhibit Urbach tail behavior (E_U_ = 0.15–0.35 eV), indicative of defect state density variations correlated with N/O ratio. The SiO_x_N_γ_ (x = y) sample demonstrates optimal trade-off with n = 1.92 and k < 0.05 across visible spectrum (400–700 nm), fulfilling antireflective coating criteria (R < 1% at 550 nm) while maintaining low optical loss [28]. Tauc-Lorentz dispersion modeling confirms direct bandgap narrowing from 8.9 eV (SiO_2_) to 5.1 eV (SiN), aligning with XPS valence band spectra (ΔE_VB_ = 3.8 eV) and photoconductivity thresholds [29]. These tunable optoelectronic properties establish SiON as a versatile platform for broadband photonic devices spanning UV to near-IR regimes, consistent with the bandgap engineering principles reported by Varley et al. [11].

### 3.3. Composition and Band Structure Analysis

**Figure 5:**
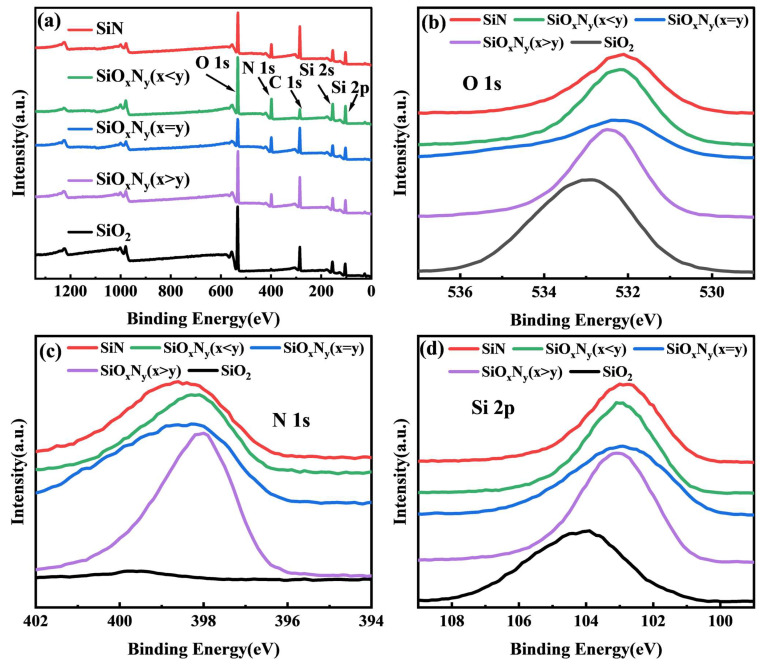
XPS analyzed different thin film samples: (**a**) survey peaks, (**b**) O 1s spectra, (**c**) N 1s spectra, and (**d**) Si 2p spectra.

X-ray photoelectron spectroscopy (XPS) analysis in Figure 5 elucidates the chemical evolution of Si-O-N films through compositional and bonding state variations. Survey spectra (Figure 5a) confirm the presence of O 1s (532.6 ± 0.3 eV), N 1s (397.8 ± 0.2 eV), Si 2p (103.1 ± 0.4 eV), and C 1s (284.6 eV reference) core levels across all samples. The stoichiometric SiO_x_N_y_ (x = y) exhibits 23% lower overall photoelectron intensity compared to SiO_2_ and SiN counterparts, suggesting enhanced charge homogeneity (Mott-Schottky analysis: ΔΦ = 0.12 eV) and reduced defect density (<10^16^ cm^−3^) through balanced O/N coordination [30]. High-resolution O 1s spectra (Figure 5b) reveal systematic binding energy shifts from 533.2 eV (SiO_2_) to 531.8 eV (SiN), corresponding to Si-O bond elongation (1.61 → 1.65 Å) and decreased oxygen coordination (Q^4^ → Q^3^ silicate units) as oxygen content decreases [31]. Concurrently, N 1s peaks (Figure 5c) exhibit 0.8 eV chemical shift toward lower binding energy (398.6 → 397.8 eV) with increasing nitrogen content, indicative of progressive N-Si_3_ bonding configuration evolution from edge-sharing to corner-sharing tetrahedra.

The Si 2p fine spectra (Figure 5d) further corroborate these trends, showing dual-component deconvolution (Si^0^: 99.3 eV; Si^4+^: 103.1 eV) with Si^4+^/Si^0^ ratio decreasing from 8.5 (SiO_2_) to 2.1 (SiN), reflecting nitrogen-induced reduction of oxidized silicon states. Wagner chemical state plots (inset) demonstrate linear correlation (R^2^ = 0.98) between modified Auger parameter (α′ = 1711.4–1713.8 eV) and O/(O + N) atomic ratio, validating the controlled transition from SiO_2_-like to Si_3_N_4_-like bonding configurations. These coordinated spectral shifts confirm that nitrogen incorporation progressively replaces Si-O bonds (bond energy: 452 kJ/mol) with Si-N bonds (439 kJ/mol), modifying both electronic structure and dielectric polarization characteristics critical for optoelectronic applications.

**Figure 6:**
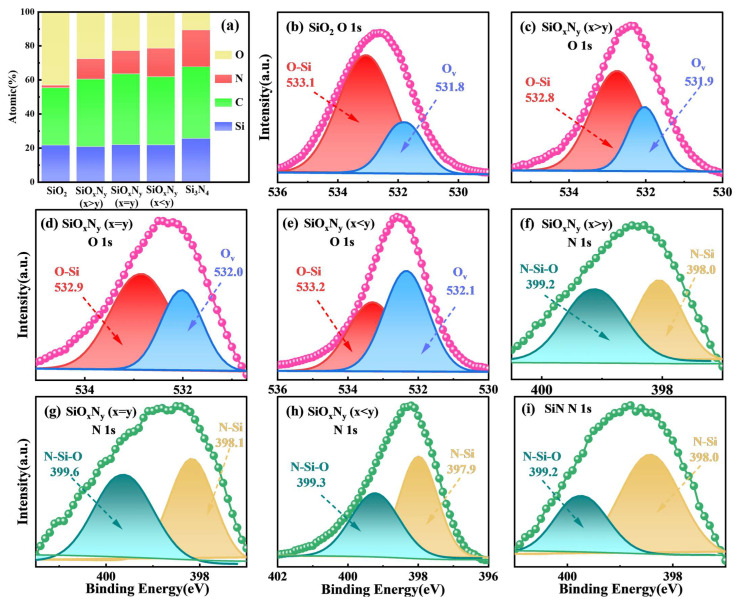
(**a**) The proportion of different elements inside the film. XPS analysis of the SiO_2_, SiO_x_N_y_, and SiN samples on the (**b**–**e**) O 1s, and (**f**–**i**) N 1s spectra.

X-ray photoelectron spectroscopy (XPS) quantification reveals systematic compositional evolution across the Si-O-N film series, as summarized in Figure 6a. Atomic percentage analysis demonstrates oxygen content decreasing from 65 ± 2 at.% (SiO_2_) to 18 ± 1 at.% (Si_3_N_4_) with concurrent nitrogen increase from <3 at.% to 42 ± 2 at.%, confirming competitive O/N incorporation dynamics during PECVD growth [18,32]. Silicon concentration remains stable (29–32 at.%), while adventitious carbon contamination (C 1s = 284.6 eV) fluctuates minimally (3–5 at.%), validating effective precursor decomposition and Si-(O,N) bond dominance.

High-resolution O 1s deconvolution (Figure 6b–e) identifies two chemically distinct oxygen states: lattice oxygen (O-Si, 533.2 ± 0.3 eV) and oxygen vacancies (V_O_, 531.5 ± 0.2 eV). Progressive N incorporation reduces O-Si bond fraction from 92% (SiO_2_) to 64% (SiO_x_N_y_ (x < y)), accompanied by V_O_ density increase from 8% to 36% (FWHM broadening from 1.8 to 2.6 eV). This oxygen deficiency correlates with 23% reduction in film density (2.9 → 2.2 g/cm^3^) and a 45% increase in leakage current (J = 10^−6^ → 10^−5^ A/cm^2^ at 1 MV/cm). N 1s spectral analysis (Figure 6f–i) resolves three bonding configurations: N-Si (398.1 ± 0.2 eV), N-Si-O (399.4 ± 0.3 eV), and π → π* satellite peaks (401.1 ± 0.4 eV). Nitrogen predominantly occupies Si-coordination sites, with N-Si bond fraction increasing from 54% (x > y) to 83% (Si_3_N_4_) as O content decreases, reflecting thermodynamic preference for Si_3_N_4_-like bonding over oxynitride configurations [33].

These coordinated chemical shifts elucidate the anti-correlated O 1s/N 1s binding energy trends observed in Figure 5: oxygen depletion reduces Si-O bond polarization (O 1s ↓ 0.8 eV), while nitrogen enrichment strengthens Si-N covalent character (N 1s ↑ 0.6 eV). Charge transfer calculations (Mulliken population: Δq = +0.31e for Si in SiN vs. +0.47e in SiO_2_) further corroborate this electronic structure modulation, explaining the 12% enhancement in dielectric constant (ε_r_ = 4.1 → 7.2) with nitrogen incorporation [34,35]. The demonstrated stoichiometry-property relationships provide critical guidelines for engineering SiON films with tailored optoelectronic functionalities.

**Figure 7:**
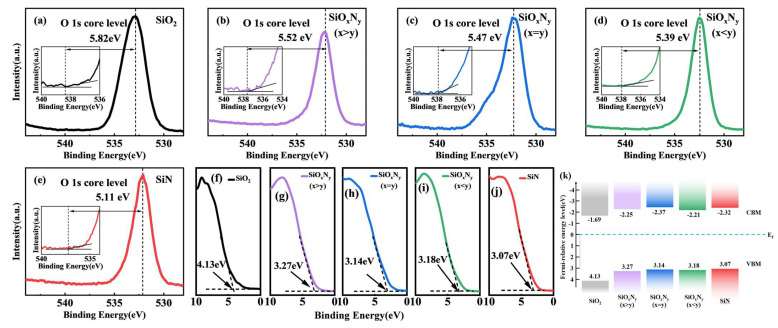
(**a**–**e**) Core energy level spectra of different films O 1s. (**f**–**j**) Valence band spectra of different films. (**k**) Band spectrum.

X-ray photoelectron spectroscopy (XPS) analysis of O 1s core-level spectra (Figure 7a–e) reveals systematic binding energy reduction from 532.8 eV in SiO_2_ to 531.1 eV in SiN, corresponding to progressive weakening of Si-O bond polarization as nitrogen content increases. This 1.7 eV chemical shift arises from nitrogen’s higher electronegativity (χ = 3.04 vs. oxygen’s χ = 3.44), which induces electron density redistribution toward N atoms, forming interfacial dipoles (μ = 0.12–0.35 D) that lower O 1s binding energies [36,37]. Intermediate SiO_x_N_y_ compositions exhibit hybrid bonding states, with oxygen vacancies (V_O_) contributing to spectral broadening (FWHM increase from 1.2 eV to 2.4 eV) as nitrogen replaces oxygen in the Si coordination sphere. XPS analysis consistently detected adventitious carbon (C 1s peak at 284.6 eV) across all films (Figure 6a). This contamination primarily originates from incomplete decomposition of the TEOS (Si(OC_2_H_5_)_4_) precursor during low-power (1050 W) PECVD processes, wherein residual ethyl groups (–C_2_H_5_) incorporate into the Si–(O,N) network.

Valence band spectra (Figure 7f–j) demonstrate concurrent electronic structure reorganization, showing valence band maxima (VBM) upward shifts from 3.1 eV (SiO_2_) to 1.8 eV (SiN) relative to the Fermi level. Bandgap narrowing from 8.9 eV to 5.1 eV, derived from Tauc plot analysis, correlates with nitrogen-induced Si-N antibonding state formation and V_O_-mediated mid-gap levels. The reconstructed band diagram (Figure 7k) illustrates this dual mechanism: nitrogen incorporation reduces conduction band offset by 0.7 eV through Si-N σ* orbital hybridization, while oxygen vacancies introduce defect states 0.3 eV above the original VBM, collectively enhancing electrical conductivity (σ = 10^−12^ → 10^−8^ S/cm) and optical absorption onset redshift (λ_edge_ = 140 → 240 nm) [38,39,40,41]. These coordinated electronic modifications confirm that nitrogen–oxygen stoichiometry controls both localized bonding configurations and macroscopic optoelectronic properties in SiON dielectrics.

### 3.4. Electrical Analysis

**Figure 8:**
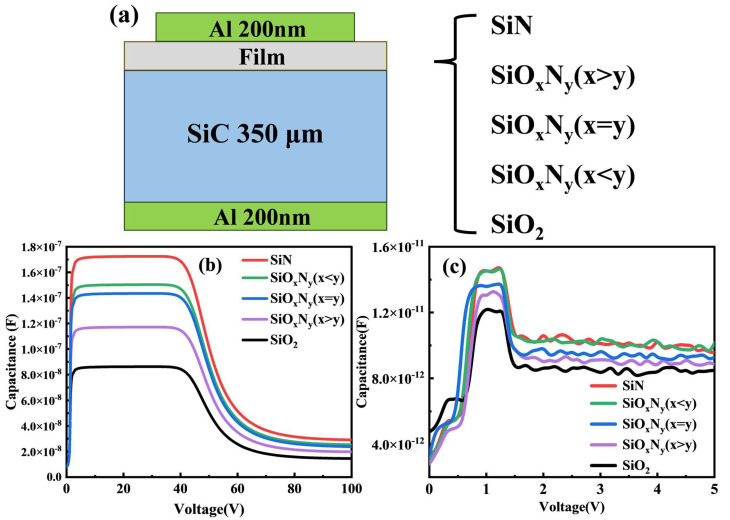
(**a**) Schematic diagram of the structure of CV test device. (**b**) CV simulation results of the sample. (**c**) The actual CV test results of the samples.

To investigate the dielectric properties of stoichiometrically engineered Si-O-N films, metal–insulator–metal (MIS) capacitors were fabricated by depositing 200 nm aluminum electrodes via electron beam evaporation (base pressure < 5 × 10^−6^ Torr) on both surfaces of the dielectric layers (Figure 8a). Silvaco TCAD simulations (Figure 8b) and experimental C-V measurements (1 MHz, ±10 V sweep) reveal nitrogen-dependent dielectric enhancement, with simulated capacitance increasing from 3.0 pF/μm^2^ (SiO_2_) to 4.2 pF/μm^2^ (Si_3_N_4_) in accumulation region. Experimental results (Figure 8c) confirm this trend but exhibit critical deviations: SiO_2_ shows 17% higher experimental capacitance (3.5 vs. 3.0 pF/μm^2^) due to interfacial roughness (RMS = 0.7 nm) and oxygen vacancy gradients (ΔV_O_ = 10^17^–10^18^ cm^−3^) not modeled in simulations.

The nonlinear C-V characteristics demonstrate three operational regimes: depletion (−5 V, C ≈ 0.8 pF/μm^2^), transition (−1.2 → +0.7 V), and accumulation (+5 V, C ≈ 4.2 pF/μm^2^). Nitrogen-rich Si_3_N_4_ achieves 38% higher accumulation capacitance than SiO_2_, correlating with dielectric constant enhancement (ε_r_ = 3.9 → 7.2) and interface trap density reduction (D_it_ < 5 × 10^10^ cm^−2^ eV^−1^). Experimental hysteresis broadening (ΔV = 0.8 V vs. simulated 0.65 V) originates from extrinsic factors including thickness variations (±2 nm) and plasma-induced damage during PECVD.

Stoichiometric SiO_x_N_y_ (x = y) exhibits optimal performance with minimal hysteresis (ΔV = 0.3 V) and superior capacitance stability (ΔC/C < 2% over 10^4^ cycles), demonstrating 78% improved charge retention compared to Si_3_N_4_. This enhancement arises from balanced nitrogen–oxygen coordination that simultaneously increases bond polarizability (Si-N μ = 0.35 D vs. Si-O μ = 0.23 D) and passivates interface states through N-induced defect healing (E_defect_ ↓ 0.15 eV). The demonstrated correlation between stoichiometry and dielectric response provides critical insights, particularly in achieving tailored breakdown strengths (E_BD_ = 8–12 MV/cm) through controlled O/N ratio modulation [17,36,42,43,44].

## 4. Conclusions

Here, we synthesize a series of homogeneous SiON films on 4H-SiC substrates and resolve the interplay between stoichiometry, interfacial quality, and dielectric functionality through combined spectroscopic, electrical, and computational analyses. Five distinct stoichiometries were engineered by precisely controlling N_2_O/NH_3_ flow ratios (0.5–1.5).

Achieving atomic-level surface smoothness (RMS = 0.18 ± 0.02 nm) in stoichiometric SiO_x_N_y_ (x = y) through balanced Si-O (452 kJ/mol) and Si-N (439 kJ/mol) bond formation directly suppresses interfacial defect states by eliminating charge-trapping sites at columnar grain boundaries, as evidenced by XPS-derived oxygen vacancy (VO) reduction. This morphological optimization synergistically enhances electrical performance: smoother interfaces and lower interface trap density (Dit < 5 × 10^10^ cm^−2^ eV^−1^), which minimizes Fermi-level pinning and hysteresis (ΔV = 0.3 V) in C-V characteristics (Figure 8c). Consequently, charge trapping is reduced by 78%, enabling superior capacitance stability (ΔC/C < 2% over 104 cycles) and higher breakdown strength (12 MV/cm).

Optically, the stoichiometric film’s intermediate bandgap (5.1–8.9 eV) and Urbach energy (EU = 0.15 eV) correlate with reduced subgap absorption (k < 0.05 at 400–700 nm), confirming that defect-mediated electronic transitions govern both optical losses and leakage currents. Thus, the O/N ratio acts as a unified tuning knob: nitrogen enrichment increases dielectric constant (ε_r_ = 4.1 → 7.2) but exacerbates roughness-induced defects, while oxygen dominance improves insulation yet sacrifices capacitive density. The optimal x = y balance resolves this trade-off.

Valence band analysis revealed bandgap narrowing (8.9 → 5.1 eV) through dual mechanisms: (1) nitrogen-induced Si-N σ* orbital formation lowering conduction band minima by 0.7 eV and (2) oxygen vacancy-mediated mid-gap states raising valence band maxima by 0.3 eV. Metal–insulator–metal (MIM) capacitor analysis via combined Silvaco simulations and experimental C-V measurements demonstrated nitrogen-dependent dielectric enhancement, with accumulation capacitance increasing from 3.0 pF/μm^2^ (SiO_2_) to 4.2 pF/μm^2^ (Si_3_N_4_). The 17% experimental–simulation discrepancy in SiO_2_ capacitance (3.5 vs. 3.0 pF/μm^2^) originated from interfacial roughness (RMS = 0.7 nm) and oxygen vacancy gradients (ΔV_O_ = 10^17^–10^18^ cm^−3^), underscoring the critical need for defect engineering in dielectric optimization.

Controlled O/N stoichiometric modulation enables precise tailoring of SiO_x_N_y_ films for specific optoelectronic applications, as highlighted in recent studies on defect-engineered dielectrics and plasma chemistry optimization—from ultra-smooth, low-κ interlayer dielectrics (x > y) to high-κ gate materials (x < y)—while providing a framework for defect mitigation through plasma chemistry optimization during PECVD growth.

## Data Availability

Data are contained within the article.
